# Mitochondrial complex I dysfunction alters the balance of soluble and membrane-bound TNF during chronic experimental colitis

**DOI:** 10.1038/s41598-022-13480-y

**Published:** 2022-06-15

**Authors:** Ainize Peña-Cearra, Miguel Angel Pascual-Itoiz, Jose Luis Lavín, Miguel Fuertes, Itziar Martín-Ruiz, Janire Castelo, Ainhoa Palacios, Diego Barriales, Asier Fullaondo, Ana M Aransay, Hector Rodríguez, Juan Anguita, Leticia Abecia

**Affiliations:** 1 CIC bioGUNE, Basque Research and Technology Alliance (BRTA), Bizkaia Science and Technology Park, Building 801A, 48160 Derio, Spain; 2 Department of Immunology, Microbiology and Parasitology, Faculty of Medicine and Nursing, University of the Basque Country (UPV/EHU), 48080 Bilbao, Spain; 3 Applied Mathematics Unit, NEIKER-Basque Institute for Agricultural Research and Development, Basque Research and Technology Alliance (BRTA), Bizkaia Science and Technology Park, Building 812L, 48160 Derio, Spain; 4 Animal Health Department, NEIKER-Basque Institute for Agricultural Research and Development, Basque Research and Technology Alliance (BRTA), Bizkaia Science and Technology Park, Building 812L, 48160 Derio, Spain; 5 Department of Genetics, Physical Anthropology and Animal Physiology, University of the Basque Country (UPV/EHU), 48080 Bilbao, Spain; 6 CIBERehd, ISCIII, 28029 Madrid, Spain; 7 Ikerbasque, Basque Foundation for Science, 48009 Bilbao, Spain

**Keywords:** Immunology, Microbiology

## Abstract

Inflammatory bowel disease (IBD) is a complex, chronic, relapsing and heterogeneous disease induced by environmental, genomic, microbial and immunological factors. MCJ is a mitochondrial protein that regulates the metabolic status of macrophages and their response to translocated bacteria. Previously, an acute murine model of DSS-induced colitis showed increased disease severity due to MCJ deficiency. Unexpectedly, we now show that MCJ-deficient mice have augmented tumor necrosis factor α converting enzyme (TACE) activity in the context of chronic inflammation. This adaptative change likely affects the balance between soluble and transmembrane TNF and supports the association of the soluble form and a milder phenotype. Interestingly, the general shifts in microbial composition previously observed during acute inflammation were absent in the chronic model of inflammation in MCJ-deficient mice. However, the lack of the mitochondrial protein resulted in increased alpha diversity and the reduction in critical microbial members associated with inflammation, such as *Ruminococcus gnavus*, which could be associated with TACE activity. These results provide evidence of the dynamic metabolic adaptation of the colon tissue to chronic inflammatory changes mediated by the control of mitochondrial function.

## Introduction

Inflammatory bowel diseases (IBD) are a group of idiopathic, chronic, relapsing and remitting inflammatory disorders of the gastrointestinal tract. Although the etiology remains unknown, the most accepted hypothesis points to an aberrant immune response to gut microbes triggered by environmental factors in genetically susceptible hosts^1^. Ulcerative colitis (UC) is one of the two major forms of IBD. The high inter-individual variability observed within human UC patients has led to develop murine models to induce colitis that display clinical characteristics similar to those of humans with IBD and help to elucidate the connection between bacteria and the host^2^.

Among different types of chemically induced colitis models, the dextran sodium sulfate (DSS)-induced colitis model is the most common model of IBD, due to its simplicity. Depending on the concentration, duration, and frequency of DSS administration, animals can develop acute or chronic colitis^3^. Acute DSS colitis has become a useful model to study the innate immune system contribution to the development of intestinal inflammation. This model is based on short-lasting barrier alterations and self-limiting inflammation. Therefore, a mouse model of chronic colitis is required to have a better understanding of the role of the adaptive immune system during intestinal inflammation.

Methylation-controlled J protein (MCJ) is a small mitochondrial protein encoded by the *Dnajc15* nuclear gene that negatively regulates the complex I activity of the electron transport chain leading to increased complex I activity^4,5^. Although not ubiquitously present in the immune system, MCJ is expressed in macrophages^6^, pivotal cells coordinating processes in the gut. DNA methylation and the transcriptional regulator Ikaros, are the mechanisms associated with the regulation of MCJ expression. In macrophages, *MCJ* expression was selectively silenced by IFNγ, under the transcriptional regulator Ikaros^7^. In general, MCJ deficiency in DSS-induced acute colitis affected disease severity, colon mitochondrial morphology, microbial composition, and primary bile acid biosynthesis^8^. In particular, lack of MCJ leads to the upregulation of *Timp3* expression resulting in the inhibition of TACE activity (TNF-α converting enzyme) which inhibits *Tnf* and *Tnfr1* shedding from the cell membrane in the colon. In addition, MCJ expression in UC patients was also lower, indicating a relevant role of mitochondrial genes and function among active UC. However, the mechanisms that regulate MCJ expression during chronic intestinal inflammation is still unknown.

Here, we induced chronic colon inflammation in an MCJ-deficient murine model resembling IBD to study the long-term impact of the absence of this mitochondrial protein. DSS-induced chronic colitis resulted in lower disease severity independently of the genotype. Interestingly, our data indicate that the expression of *MCJ* could be regulated over the course of chronic inflammatory disorders providing the tissue with the capacity to metabolically adapt. Results point to TACE activity as a relevant factor in the interaction between the host and their microbiota.

## Methods

### Animals and experimental design

6–8 weeks old C57BL/6 J WT and MCJ-deficient female mice were used^4^. Mice received food and water ad libitum and were maintained in standard 12 h light/dark cycles. Thirty two mice were used in total (16 WT and 16 MCJ deficient mice). Chronic experimental colitis was induced to half of them by three cycles of 2% DSS that consisted of 7 days of DSS administration in the drinking water followed by 14 days of water. After the third cycle of DSS mice recovered for 3 days (1st cycle: from day 0 to day 7, 2nd cycle: from day 21 to day 28 and 3rd cycle: from day 42 to 49). A blind technician monitored daily the Disease Activity Index (DAI), which is based on the average of 3 parameters; weight loss, stool consistency and rectal bleeding. To assign DAI scores the proposed criteria by Camuesco et al.^9^, were followed (Supplementary table S1). Rectal bleeding was scored as visible blood in feces, from slightly bloody to highly bloody stools (Supplementary table S1). Spleen, mesenteric lymph nodes, colon tissue and colon content were collected at sacrifice. Then, colon lengths (cm) was measured using a standard rule and spleen weights (g) was also recorded.

### Transepithelial permeability assay

Mice were orally gavaged with 600 mg/kg of body weight of FITC–dextran (4 kDa; TdB consultancy) and whole blood was collected by cardiac puncture 4 h after gavage. Blood serum was collected after centrifugation at 6000 rpm for 10 min. Serum fluorescence intensity was measured using a multi-detection microplate reader (Spectramax M2, Molecular devices) with an excitation wavelength of 485 nm and an emission wavelength of 528 nm. FITC concentration (mg ml^−1^) was calculated from a standard curve using serial dilutions of FITC–dextran^10^.

### Myeloperoxidase activity assay

One centimeter of the distal colon was homogenized in 50 mM phosphate buffer (pH 6.0) and 0.5% hexadecyltrimethylammonium bromide (CTAB) using a Precellys 24 homogenizer. To determine myeloperoxidase activity, the detailed protocol published by Pascual-Itoiz et al. was carried out^8^.

### Histology and immunohistochemistry

Colon tissue was fixed in 10% formalin or Carnoy´s fixative, dehydrated, embedded in paraffin, and cut into 5 μm-thick sections. For histopathology, sections were deparaffined, hydrated and stained with hematoxylin and eosin or periodic acid Schiff (PAS) according to standard protocols. Stained sections were analyzed blindly by a pathologist. The number of goblet cells was determined on PAS-stained slides and expressed as percentages per intestinal epithelial cells.

Colon serial sections (5 µm) were subjected to immunohistochemistry with primary antibody specific for IgA, HRP conjugated (IgA; dilution 1:100). Antigen retrieval was performed incubating with proteinase K for 15 min at 37ºC. After incubation, tissue sections were immersed in DAB solution for 2 min and then washed. Slides were counterstained in Mayer´s hematoxylin for 30 s. Images were captured with a Zeiss Axioimager A1 microscope and analyzed with Frida software. At least 10 visual fields were captured randomly.

### Colon proteins extraction and quantification

Proteins from colonic tissue were extracted with radioimmunoprecipitation assay lysis buffer (RIPA buffer) with several phosphatase inhibitors such as sodium fluoride (1 mM), sodium orthovanadate (1 mM) and B-Glycerophosphate (1 mM) and a protease inhibitor cocktail (1 tablet/50 ml lysis buffer). Colonic tissue was grinded on Precellys tissue homogenizer. Lysate solution was centrifuged and the supernatant was stored at -80ºC.

Protein concentrations were measured with the Pierce™ Rapid Gold BCA Protein Assay Kit (Thermo Fisher Scientific) following manufacturer’s instructions.

### TACE activity assay

TACE activity from murine colon protein extracts was measured following the protocol previously published by Pascual-Itoiz et al.^8^.

### IL-10 enzyme-linked immunosorbent assay (ELISA)

IL-10 levels from colonic protein extracts were determined using the Mouse IL-10 ELISA Set (RUO) (BD Biosciences) according to the manufacturer’s recommendations.

### Cell preparation

Spleens and mesenteric lymph nodes were dissected post-mortem and collected in PBS (Gibco). For splenocyte and lymph node cell preparation, organs were mashed through a 70-μm cell strainer (Falcon), and erythrocytes from spleens were lysed using ACK (Ammonium-Chloride-Potassium) Lysis Buffer.

### FACS analysis

Two different stains were performed with fluorochrome-conjugated antibodies. The first staining consisted of CD45R (B220) PE (Miltenyi Biotech, REA755); CD4 FITC (Miltenyi Biotech, GK1.5); CD8 PerCP-Vio 700 (Miltenyi Biotech, 53–6.7); CD69 APC-Vio 770 (Miltenyi Biotech, H1.2F3); CD44 APC (Miltenyi Biotech, IM7.8.1). The second staining included CD11b APC (Miltenyi Biotech, M1/70); CD103 PE (Miltenyi Biotech, REA789); F4/80 PE-Vio 770 (Miltenyi Biotech, REA126); MHCII APC-Vio 770 (Miltenyi Biotech, REA813). Fc receptors were blocked with Anti-mCD16/CD32 (BD). Only events that appeared single in forward-scatter width were analyzed. FACSCanto II and FACSDiva software (BD) were used for flow cytometry and data were analyzed using FlowJo software (TreeStar).

### RNA extraction, cDNA synthesis and gene expression

Colon samples were collected and frozen at -80ºC. The day before the RNA extraction, 200 µl of RNAlater solution was added to the samples and stored at -20ºC. Total RNA from colon tissue was extracted using RiboZol and the Nucleospin RNA kit (Macherey–Nagel) according to the manufacturer`s protocol. M-MLV reverse transcriptase (ThermoFisher Scientific) was used to synthesize cDNA. RT-PCR was performed on 384 well plates in a QuantStudio 6 Flex Real-Time PCR system (Thermo Fisher Scientific, Waltham, MA, USA) with PerfeCTa SYBR Green SuperMix Low ROX (Quantabio, Beverly, MA, USA) and amplification was analyzed by QuantStudio RT PCR software v1.3. Primers for *Ifng*, *Il1b, Lcn2, Mcj, Myd88,Timp3, Tlr4, Tlr5, Tlr9*, *Tnf*, *Tnfr1, Reg3b* and *Rpl19* genes were optimized (see sequences and annealing temperature in supplementary table S2). To normalize mRNA expression, the expression of 3 housekeeping genes was measured and *Rpl19* was ranked as the best candidate. The mRNA relative quantification was calculated using the ΔΔCt method^11^. PCR efficiency was always between 90 and 110%.

### DNA extraction and microbiome analysis

Colon content was collected at sacrifice. DNA was isolated from freeze-dried colon samples using the FavorPrep Stool DNA Isolation Mini kit (Vienna, Austria) following the manufacturer’s instructions. Eluted DNA concentration was assessed spectrophotometrically using a NanoDrop ND-100 Spectrophotometer (NanoDrop Technologies, Wilmington, DE, USA). Purified DNA samples were stored at − 20ºC until use.

Using high throughput sequencing platforms and barcoded primer sets, *16S rRNA* gene was used to deeply characterize the microbial populations present in the colon of experimental mice. DNA extracts were used as template for PCR-based barcode amplification of the bacterial V3-V4 region of the *16S rRNA* gene using Illumina Inc. Miseq with 2 × 250 bp paired-end reads following the manufacturer´s standard protocol.

Data processing was performed using QIIME (v.1.9.0): Quantitative Insights Into Microbial Ecology software package^12^. Sequences were clustered as operational taxonomic units (OTUs) of 97% similarity using UCLUST^13^. OTUs were checked for chimeras using the RDP gold database and taxonomy was assigned using the Greengenes database (version 4feb2011)^14^. Richness (number of observed species) and alpha and beta diversity metrics (Chao1, Shannon index, and phylogenetic Diversity whole tree) were calculated using the QIIME pipeline. The significances of grouping in the PCoA plots were tested and analysis of similarity (ANOSIM) with 999 permutations. We further performed statistical analyses to detect differences in microbial composition between groups with DESeq2 package^15^ for R and Linear Discriminant Analysis Effect Size (LEfSe) tool^16^. Charts were plotted using several R packages, including Phyloseq, ggplot2, ggpubr, reshape2 and qplots. Raw sequences were deposited in the European Nucleotide Archive (ENA) under the project number PRJEB44741.

### Ethics approval

Animal protocols were approved by the Animal Research Ethics Board of CIC bioGUNE (Spain; permit number CBBA-0615) and the competent authority (Diputación de Bizkaia) in accordance with European and Spanish guidelines and regulations. ARRIVE guidelines were used to report animal experiments.

## Results

**Figure 1:**
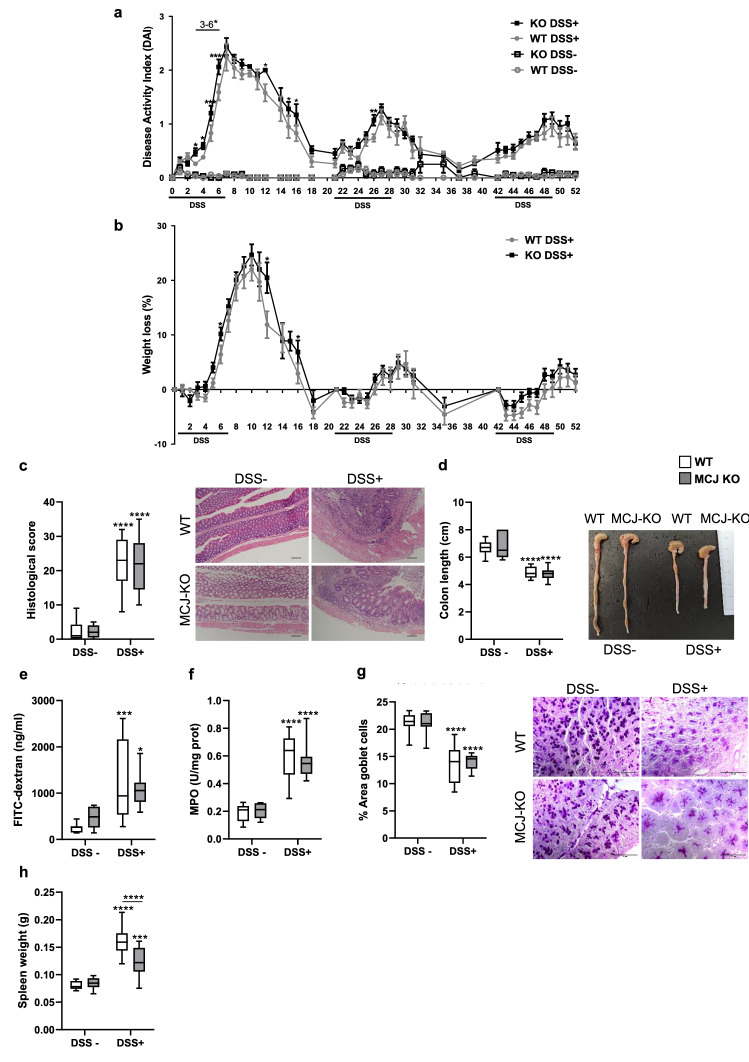
Characterization of DSS-induced chronic colitis effect in MCJ-deficient mice. To induce chronic colitis, mice received 3 cycles of 2% DSS during 7 days with 15 days with water in between. In the last cycle of DSS mice were let 3 days with water to recover. (**a)** Disease activity index and (**b)** weight loss percentage; data are means ± SEM. (**c)** Histological score and representative images of the colon tissue stained with hematoxylin and eosin (× 100). (**d)** Colon length (cm) and representative images. (**e)** Permeability to the tracer FITC-dextran (ng/ml). (**f)** Myeloperoxidase activity (U/mg prot). (**g)** Goblet cells: % of positive cells stained with PAS (Periodic Acid-Schiff) and representative images of the stained colon tissue (× 400, scale bar = 100 μm). (**h)** Spleen weight (g). Grey lines and white boxplots indicate WT and black lines and grey boxplots MCJ-KO mice. Box whisker plots of median, quartiles and range with at least 9 mice per group. For statistical analysis, two-way ANOVA was used as applicable, *P < 0.05, **P < 0.01, ***P < 0.001, ****P < 0.0001. “*” above boxes versus control genotype (DSS + *versus* DSS-), “*” above line versus different genotypes in the same experimental group.

To examine MCJ contribution to chronic colitis, disease severity parameters were assessed in healthy and chronic DSS-induced colitis WT and MCJ-deficient mice. Significant differences between genotypes in Disease Activity Index (DAI) were detected in the first cycle of DSS (days 3–6, 12, 15 and 16) and only at days 24 and 26 of the second cycle (Fig. 1a). Strikingly, no differences were observed during the third cycle of DSS. Sustained weight loss was only detected on certain days of the first cycle (days 7, 13 and 16) (Fig. 1b). At the end of the experimental period histological score, colonic length, intestinal permeability, neutrophil infiltration (MPO) and goblet cells quantity only changed due to DSS treatment (Fig. 1c-g). Spleen weights also augmented after DSS treatment, albeit MCJ-deficient mice had significantly lower spleen weights than WT mice (Fig. 1h). Altogether, these data indicate that MCJ plays a key role in acute colitis, impacting the innate immune system response but its effect fades over the course of the disease.

**Figure 2:**
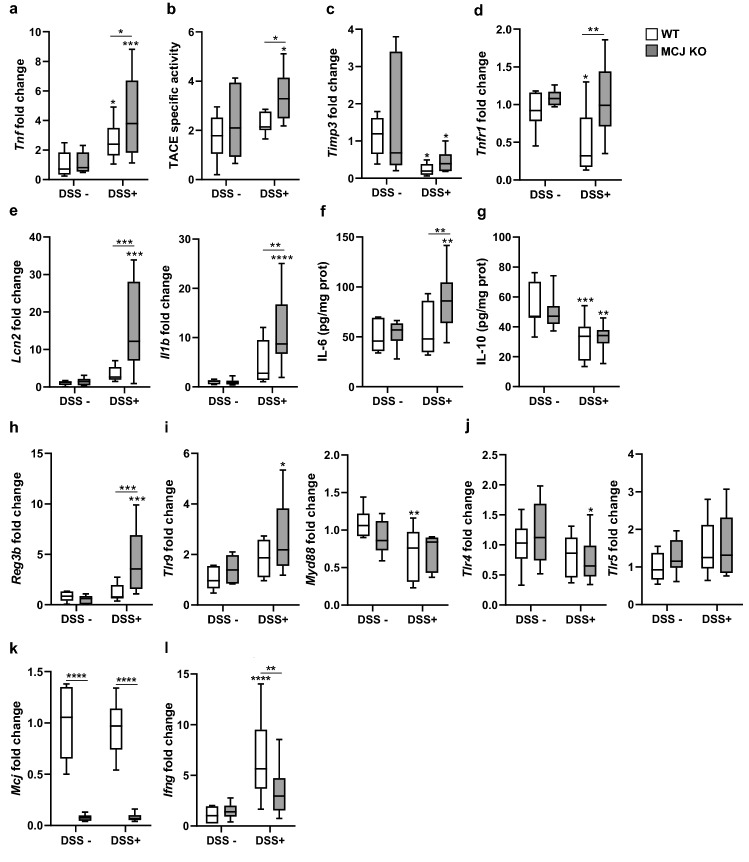
Evaluation of relevant genes and proteins during chronic colitis. Gene expression analysis from mice colon tissue of (**a**) *Tnf*, (**c**) *Timp3*, (**d**) *Tnfr1*, (**e**) *Lcn2* and *Il1b*, (**h**) *Reg3b*, (**i**) *Tlr9 and Myd88*, (**j**) *Tlr4 and Tlr5*, (**k**) *Mcj* and (**l**) *Ifng* shown as mean fold change of treated experimental groups *versus* untreated WT group (DSS-) (n = 9 mice per group at minimum). (**b**) TACE specific activity (n = 7). (**f**) IL-6 and (**g**) IL-10 ELISA (pg/mg prot). White boxplots indicate wild-type and grey boxplots MCJ-deficient mice. Box and whisker plots of median, quartiles and range. For statistical analysis two-way ANOVA was used as applicable, *P < 0.05, ** P < 0.01, ***P < 0.001, ****P < 0.0001. “*” above boxes *versus* control genotype (DSS- *versus* DSS +), “*” above line *versus* different genotypes in the same experimental group.

Next, we tested whether MCJ levels during chronic inflammation influence the inflammatory output and the TNF pathway modified due to MCJ deficiency. Of note, gene expression levels of *Tnf* substantially increased in the absence of MCJ in chronic inflammation compared to the WT genotype (Fig. 2a). Moreover, the metalloprotease TACE, involved in the cleavage of membrane-bound TNF (mTNF) to produce soluble TNF (sTNF), also increased after chronic colitis induction in MCJ-deficient mice compared to WT, suggesting a higher sTNF/mTNF ratio (Fig. 2b). In addition, no differences were observed between genotypes regarding *Timp3* levels (TACE inhibitor) in healthy controls and chronic DSS-induced colitis groups and decreased expression of *Timp3* was detected after chronic colitis induction in both genotypes (Fig. 2c). Although gene expression of the TNF receptor 1 (*Tnfr1*) did not significantly increase under chronic inflammatory conditions, MCJ-deficient DSS-treated group of mice showed higher *Tnfr1* levels than WT mice (Fig. 2d). Importantly, gene expression levels of *Lcn2,* a biomarker of intestinal inflammation released by immune cells, and *Il1b,* the potent pro-inflammatory cytokine induced during colitis, were highly affected by MCJ deficiency when mice were treated with DSS (Fig. 2e,). Notably, MCJ-deficient mice displayed higher *Il6* levels in chronic colitis compared to WT (Fig. 2f). Regarding IL-10, lower amounts were observed under inflammatory conditions in both WT and MCJ-deficient mice compared to healthy animals (Fig. 2g). Remarkably, expression of the antimicrobial *Reg3b* was only increased in MCJ-deficient mice after induction of chronic colitis suggesting a more protective environment (Fig. 2h). Although *Tlr9* expression was increased after chronic colitis induction in MCJ-deficient mice, no significant differences were detected between genotypes, and the expression of *Myd88* was not affected, suggesting that the *Myd88*-*Tlr9* signaling pathway might be critically regulated in the innate immune response, but not in chronic responses (Fig. 2i). Additionally, *Tlr4* and *Tlr5* remained unchanged in colon tissue (Fig. 2j). Next, we confirmed that DSS administration did not affect MCJ gene expression (Fig. 2k). However, the expression of *Ifng*, which is known to act as an inhibitor of MCJ gene transcription, was highly increased in WT mice upon intestinal inflammation compared to MCJ-deficient mice (Fig. 2l). Collectively, these results indicated that genes involved in the TNF pathway were significantly influenced by MCJ deficiency during chronic inflammation. Nevertheless, an opposite gene regulation of TACE and *Timp3* compared to acute colitis was found, which suggested an impact also on both forms of TNF (sTNF and mTNF).

**Figure 3:**
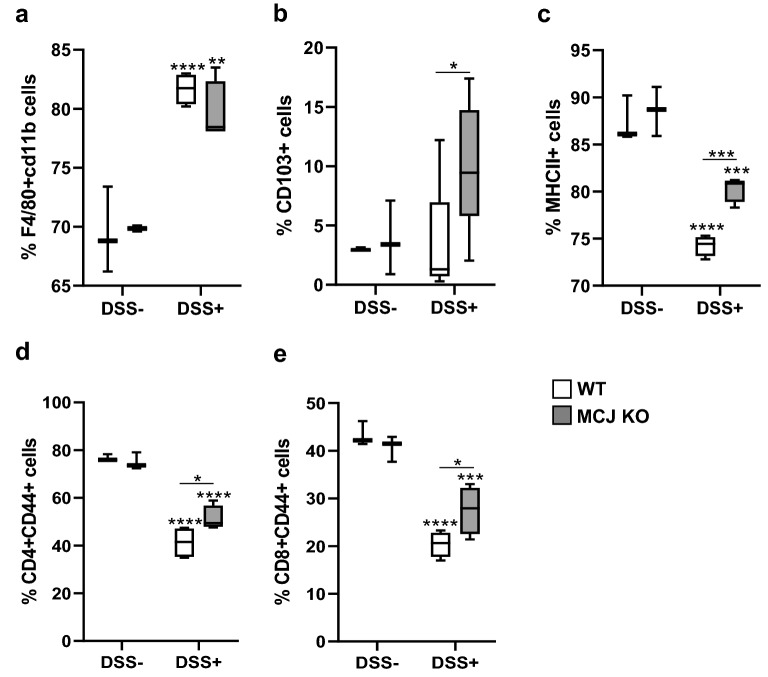
Characterization of cellular immune responses in murine DSS-induced chronic colitis model. Flow cytometry analysis of lymphoid populations in mesenteric lymphoid nodes, shown as percentage. (**a**) F4/80 + CD11b macrophages, (**b**) CD103 + dendritic cells, (**c**) MHCII + antigen presenting cells, (**d**) CD4 + CD44 + effector/memory T and (**e**) CD8 + CD44 + effector/memory T cells. Statistical differences. Box and whisker plots of median, quartiles and range, n = 4 mice per group at a minimum. For statistical analysis two-way ANOVA was used as applicable, *P < 0.05, **P < 0.01, ***P < 0.001, ****P < 0.0001. “*” above boxes *versus* control genotype (DSS- *versus* DSS +), “*” above line *versus* different genotypes in the same experimental group.

Then, we sought to clarify the role of MCJ in innate and adaptive immune responses in DSS-induced chronic colitis. Therefore, we determined the number and activation status of innate and adaptive cells in the mesenteric lymph nodes (MLNs). Within macrophages, significantly higher percentages of F4/80 + CD11b + cells were observed in mice administered with DSS compared to untreated mice regardless of the genotype (Fig. 3a). However, the percentage of CD103 + dendritic cells (DCs) increased in the absence of MCJ upon DSS treatment (Fig. 3b).

We also analyzed markers of immune cell activation in the MLNs. The treatment with DSS resulted in a decreased percentage of MHC II + cells (Fig. 3c). However, even though they were also reduced compared to non-treated controls, the percentage of MHC II + cells was significantly higher in MCJ-deficient mice, compared to controls (Fig. 3c). Furthermore, the percentage of CD44 + cells in both the CD4 + and CD8 + T cell compartments were significantly higher in the absence of MCJ than in WT mice, albeit with a significant reduction in both genotypes compared to untreated animals (Fig. 3d, e), probably as a result of T cell mobilization from the MLN to the lamina propria.

**Figure 4:**
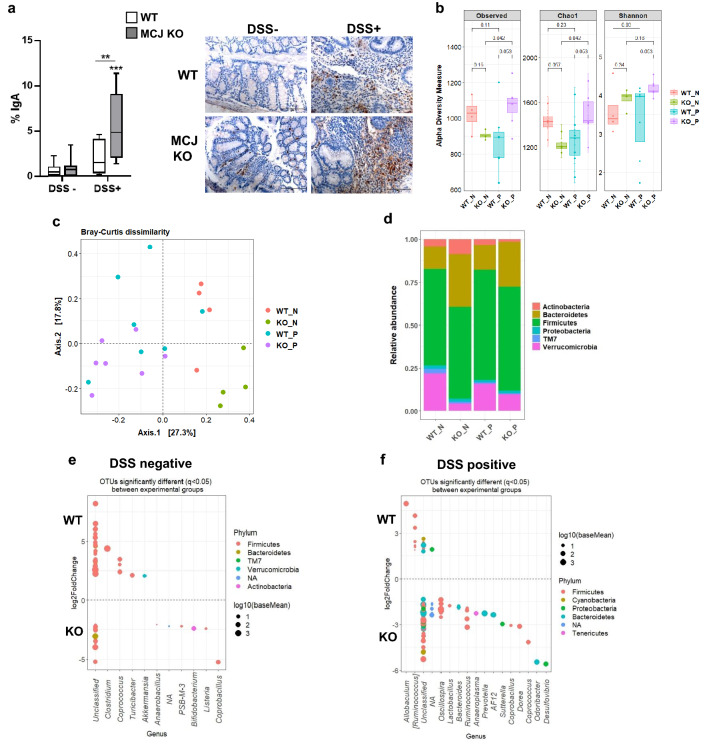
Composition of host microbiome after chronic-induced colitis. (**a**) Quantification and representative images of IgA levels in colon tissue by immunohistochemistry (× 400, scale bar = 100 μm). Box and whisker plots of median, quartiles and range, n = 8 mice per group at a minimum. For statistical analysis two-way ANOVA was used as applicable, *P < 0.05, **P < 0.01, ***P < 0.001, ****P < 0.0001. “*” above boxes versus control genotype (DSS- *versus* DSS +), “*” above line versus different genotypes in the same experimental group. (**b**) Alpha diversity (total observed taxonomic units, Chao1 and Shannon diversity indices) in mice fecal microbiota. The non-parametric Wilcoxon rank sum test was used for the statistical analysis. (**c**) Principal Coordinates Analysis (PCoA) plot of bacterial beta-diversity based on Bray–Curtis dissimilarities. (**d**) Stacked bar plots showing the average relative abundance of bacterial phyla in the different experimental groups. Representation of OTUs that significantly (q < 0.05) differ in colon content at genus level between (**e**) WT and MCJ-deficient controls and (**f**) WT and MCJ-deficient chronic colitis groups. Each point represents a single OTU colored by phylum and grouped by taxonomic family, and point´s size reflect the mean abundance of the sequenced data.

Secretory IgA provides protection against pathogens and toxins through immune exclusion and binds to bacteria regulating the composition and function of gut microbiota. Therefore, IgA levels were measured and a marked increase of IgA was found in chronic colitis-induced MCJ-deficient mice compared to WT animals (Fig. 4a). Increased IgA responses in the absence of MCJ could be associated with changes in the intestinal microbiota.

Then, the bacterial *16S rRNA* gene was evaluated. A high taxonomic resolution was obtained with mean read counts of 97,954 ± 19,769 sequences per sample and a Good´s coverage percentage of 99%. First, microbial alpha diversity analysis revealed microbiota stabilization after 3 cycles of DSS, in fact, diversity indices did not show a substantial reduction due to DSS administration, which is typically observed in acute colitis (Fig. 4b). Remarkably, alpha diversity indices (Observed species and Chao1) significantly increased in MCJ-deficient mice when they were treated with DSS (*p* value = 0.04). The principal coordinate analysis (PCoA) based on Bray–Curtis distances depicted patterns from gut microbial communities of healthy and chronic colitis WT and MCJ-deficient mice. The non-parametric statistical method ANOSIM did not show significant differences between WT and MCJ-deficient mice microbial communities, either in homeostasis or chronic colitis (Fig. 4c). Nevertheless, WT and MCJ-deficient mice exhibited distinct microbial composition in homeostasis at the phylum level (Fig. 4d). Untreated MCJ-deficient mice presented higher Actinobacteria (8.4% vs 4.4%) and Bacteroidetes (31.1% vs 12.6%) compared to healthy WT. Moreover, we observed a significant enrichment of the phylum Verrucomicrobia in WT mice compared to MCJ-deficient mice (21.8% vs 4.8%). Of note, among all the significant differences identified at the phylum level in homeostasis, after chronic colitis induction only the differences observed at Bacteroides level persisted. To determine the OTUs that were statistically different between WT and MCJ-deficient gut microbial communities, the DESeq2 tool was used. Without DSS administration, WT mice displayed increased levels of *Akkermansia*, *Clostridium*, *Coprococcus* and *Turicibacter* genera whereas MCJ-deficient mice showed higher levels of *Anaerobacillus*, *Bifidobacterium*, *Coprobacillus and Listeria* (Fig. 4e). After DSS induction, *Ruminococcus gnavus*, commonly found in the absence of MCJ during acute inflammation, and the *Allobaculum* genus was significantly enriched in WT chronic colitis, compared to MCJ-deficient condition (Fig. 4f). Noteworthy, the abundance of *R. gnavus* augmented in WT (WTp) mice during chronic inflammation compared to healthy WT (WTn) (Supplementary Fig. S1a). Nonetheless, MCJ-deficient mice exhibited enriched abundance of many genera compared to WT upon chronic inflammation, including *Bacteroides*, *Coprobacillus*, *Coprococcus*, *Desulfuvibrio*, *Dorea*, *Lactobacillus*, *Oscillospira*, *Prevotella* and *Suterella* (Fig. 4f). Furthermore, specific bacterial differences were also observed between MCJ-deficient groups (Supplementary Fig. S1b). Altogether, these data suggest that induction of chronic colitis diminishes microbial composition differences between genotypes observed during acute colitis model. In addition, results point to specific members of the gut community as critical ones contributing to the disease severity. The high levels of IgA observed in the absence of MCJ could be partially responsible for the shifts in bacterial composition.

## Discussion

Acute models of IBD have been widely used to reduce the heterogeneity observed in humans and to analyze the onset of the disease characterized by a higher contribution of the innate immune system. However, human IBD is a chronic relapsing and remitting disorder that involves chronic inflammation of the gastrointestinal tract. Therefore, a mouse model of chronic colitis was used to assess the contribution of MCJ in adaptive immunity.

In our previous work, the MCJ-deficient murine model of DSS-induced acute colitis showed inhibited TACE activity and increased *Timp3* expression levels, which effectively prevented the shedding of TNF from the plasma membrane. Thus, the loss of MCJ resulted in increased transmembrane TNF, which could lead to a higher disease severity^8^. Conversely, within chronic inflammation, we observed an augmented TACE activity in MCJ-deficient mice and *Timp3* depleted in both genotypes. Therefore, our results suggested a higher sTNF/mTNF ratio in MCJ-deficient mice in this condition. This is in accordance with previous results from acute model of colitis where TACE activity was affected probably due to colon microbial composition selected by MCJ deficiency^8^. Therefore, data suggested that the distinct microbiota composition observed in chronic colitis compared to acute colitis could be responsible for this effect. Despite being both forms of TNF detrimental for the course of colitis, the expression of mTNF by CD14 + macrophages has been reported to be relevant in IBD^17^, and mTNF signaling has been implicated in the development of granulomas in CD patients^18^. Furthermore, inflammation could not be reduced by neutralization of sTNF, which suggested that the development and maintenance of inflammation might be dependent on mTNF^18^. Besides the strong evidence of TNF as a pro-inflammatory cytokine, TNF has also immunosuppressive functions that might play a beneficial role in IBD. In this regard, sTNF has been shown to sensitize activated T cells for apoptosis during the priming phase, thereby attenuating the extent and duration of T-cell reactivity, and consequently limiting T-cell-mediated inflammation^19^. Collectively, our data suggest that the form of TNF highly affects disease severity during chronic colitis, and that sTNF could be linked to the amelioration of the MCJ-deficient phenotype reported in acute colitis.

The high levels of IL-1β secreted by colon lamina propria monocytes have been associated with disease activity and the high expression of *LCN2* by gut epithelial cells has been found in the inflamed areas of patients with IBD^20–22^. Thus, both proteins serve as valuable indicators of inflammation. In agreement with our results, *Il1b* and *Lcn2* increased in MCJ-deficient mice upon chronic inflammation. Indeed, results agreed with augmented *Il1b* reported in acute colitis due to MCJ deficiency^8^. Nevertheless, we did not observe the increased expression of the *Tlr9*/*Myd88* pathway shown within acute colitis due to MCJ deficiency^8^. In this regard, *Tlr9* can respond to mitochondrial DNA, which is commonly released during active IBD, contributing to inflammation^23^. Hence, although the expression of pro-inflammatory cytokines was affected by MCJ level in colon tissue, it was not strong enough to affect disease severity.

Antigen presenting DCs and macrophages are inducers and regulators of immune responses. Although we did not detect differences between genotypes regarding macrophage infiltration upon chronic inflammation, a significant influx of DCs into the MLN in MCJ-deficient mice was observed, which was opposed to acute colitis. Even though DSS colitis is caused primarily by disruption of the epithelium and activation of macrophages and neutrophils, T-cell responses affect the inflammatory response at later phases of the disease. During chronic DSS-induced colitis, T cells might play an important role and interfere with colonic healing^24^. Remarkably, it was found a decline in both activated (CD44 +) T CD4 and T CD8 cells in the MLN of mice with chronic colitis compared to controls, suggesting their mobilization to the lamina propria. However, after induction of chronic inflammation, MCJ-deficient mice displayed a higher percentage of these cells. These T-cell responses can result in effector cytokine production that contributes to the high levels of TNF and IL-6 produced by innate cells in both forms (acute and chronic) of DSS colitis. Although IL-6 is linked to the initiation of pro-inflammatory responses, recent evidence also suggests its involvement in healing processes^25^. Moreover, IL-6 has a large impact on antigen-specific B cell responses that could result in a large number of plasma cells in the lamina propria leading to higher IgA levels^26^. In this regard, a recent study showed that the altered microbiota of mice deficient in the kinase TAK1, promoted both IL-1β and IL-6 signaling pathways that are needed for the induction of protective intestinal Th17 cells^27^. Overall, higher production of IL-6 and IL-1β in MCJ-deficient mice could indicate a higher presence of protective T cells, and therefore, resistance to colitis.

Changes in gut microbiota composition due to mitochondrial dysfunction are a hallmark in the pathogenesis of UC. Nevertheless, the differences observed in gut microbiota composition from healthy to colitis or between genotypes during acute colitis disappeared in the chronic model of ulcerative colitis, probably as a result of microbiota-host adaptation to multiple cycles of DSS to induce the chronic inflammation. Despite being microbiota composition characterized by increased levels of Proteobacteria, both genotypes showed low quantities of Proteobacteria upon chronic inflammation in agreement with Xu et al.^28^. They concluded that the changes reported in UC patients were similar to those observed in the acute colitis model, and not in the chronic colitis model. It seems that DSS-induced chronic colitis mice might become partially adapted to the inflammatory environment independently of the initial level of MCJ. Furthermore, it was demonstrated that the expression of MCJ in macrophages could be regulated to adapt metabolically to new conditions, as it could be the case in a chronic inflammation^7^.

Host–microbial interactions are critical in the disease. Therefore, some of the shifts observed due to MCJ deficiency, including increased *Lcn2*, *Reg3b* and IgA, could be implicated in the regulation of gut microbial composition mirroring microbiota in the control group (WT). Among the diverse physiological functions of *Lcn2*, one is to limit bacterial growth enhancing their efficacy as a treatment for experimental colitis. *Reg3b* modulates host defense processes via bactericidal activity on Gram-positive bacteria. In addition, augmented levels of IgA could be associated with a higher binding to gut microbiota that regulates its composition and function^29^. Hence, these shifts could have shaped microbiota composition in the absence of MCJ, resulting in decreased *R. gnavus* levels compared to WT animals and improving the disease outcome.

Collectively, results from the present work provide evidence of the dynamic adaptation of the colon tissue to changes in the environment as a way to adapt metabolically to inflammatory conditions. Our results show that the control of *MCJ* expression impacts on TACE activity and consequently alter the balance between soluble and membrane-bound TNF, constituting a mechanism to regulate cellular responses to environmental changes during chronic ulcerative colitis. In addition, specific microbial changes due to MCJ deficiency may also be associated with the TACE activity confirming the important role of this factor in the microbiota host interaction and disease progression. Overall, it can be inferred that the role that MCJ deficiency plays between microbiota and the host during acute colitis changed after repeated cycles of DSS, probably due to the mutual adaptation to the chronic inflammatory environment.

## Supplementary Information

Supplementary Information 1.

Supplementary Information 2.

## Data Availability

Raw sequences used for metagenomics analysis were uploaded to European Nucleotide Archive (ENA www.ebi.ac.uk/ena) under the project number PRJEB44741.
