# Supplementary material for: Mitochondrial complex I dysfunction alters the balance of soluble and membrane-bound TNF during chronic experimental colitis
Source: Sci Rep. 2022 Jun 15;12:9977. doi: 10.1038/s41598-022-13480-y (PMC9200762; doi:10.1038/s41598-022-13480-y)
Supplement: Supplementary file 1 — Supplementary Information 1. [file 41598_2022_13480_MOESM1_ESM.pptx]

## Slide 1
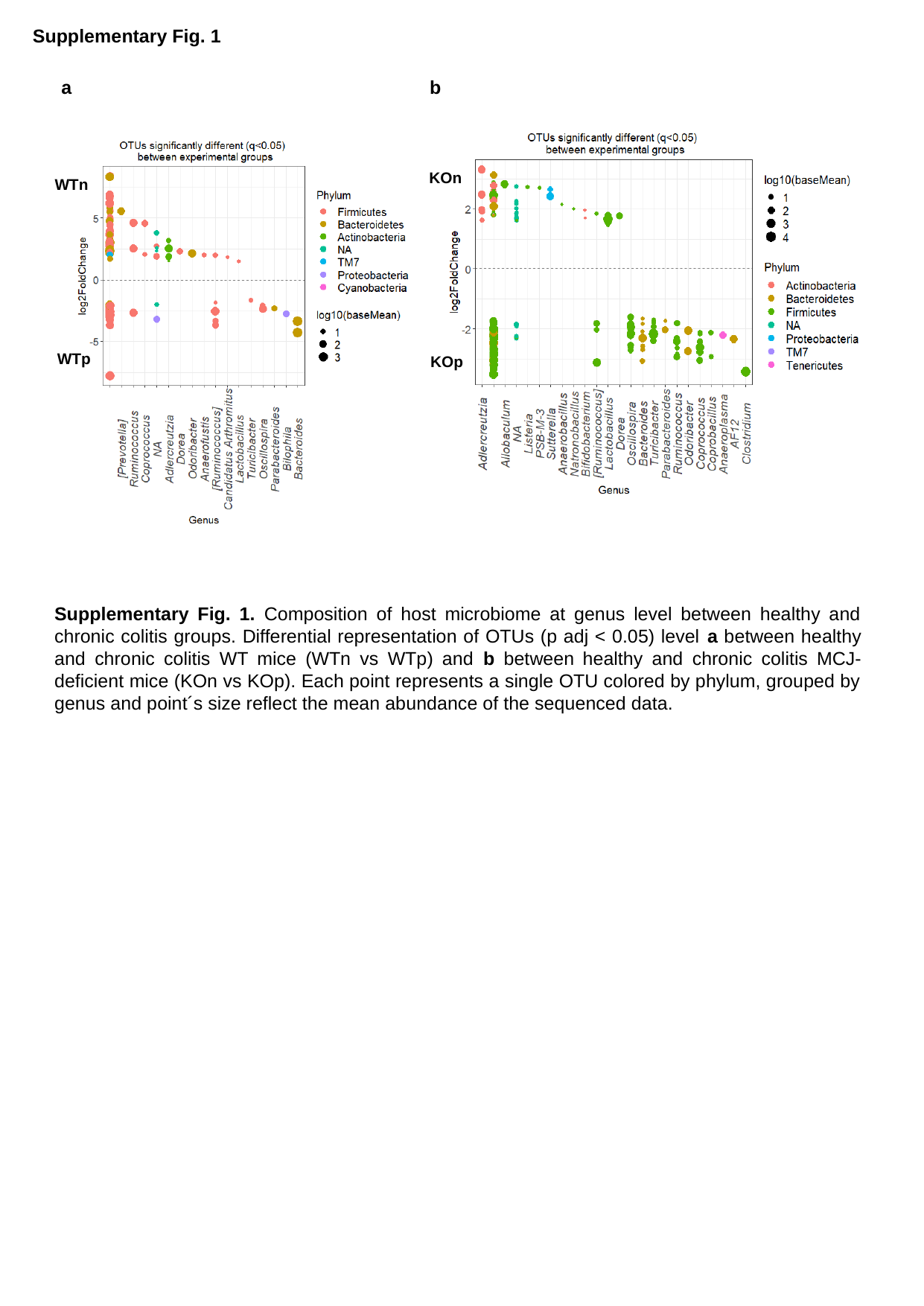

Supplementary Fig. 1
b
a
KOn
KOp
WTn
WTp
Supplementary Fig. 1. Composition of host microbiome at genus level between healthy and chronic colitis groups. Differential representation of OTUs (p adj < 0.05) level a between healthy and chronic colitis WT mice (WTn vs WTp) and b between healthy and chronic colitis MCJ-deficient mice (KOn vs KOp). Each point represents a single OTU colored by phylum, grouped by genus and point´s size reflect the mean abundance of the sequenced data.
