# Supplementary material for: Mitochondrial complex I dysfunction alters the balance of soluble and membrane-bound TNF during chronic experimental colitis
Source: Sci Rep. 2022 Jun 15;12:9977. doi: 10.1038/s41598-022-13480-y (PMC9200762; doi:10.1038/s41598-022-13480-y)
Supplement: Supplementary file 2 — Supplementary Information 2. [file 41598_2022_13480_MOESM2_ESM.docx]

**Supplementary Table 1. Disease Activity Index scoring system.** DAI index was calculated as total score of the sums of body weight lost, stool consistency and rectal bleeding divided by 3. Table adapted from Camuesco et al.^9^.

| **Score** | **Weight loss (%)** | **Stool consistency** | **Visible fecal blood** |
| --- | --- | --- | --- |
| 0 | None | Normal | Normal |
| 1 | 1-5 |  |  |
| 2 | 5-10 | Loose stools | Slight bleeding |
| 3 | 10-20 |  |  |
| 4 | >20 | Diarrhea | Gross bleeding |

**Supplementary Table 2.** Forward and reverse primer sequences (5´ to 3´), annealing temperature (T^a^) and the purpose of the mouse primers used for murine qPCR analysis.

| Gene | Forward (F) and reverse (R) primer sequences (5´- 3´), | T^a^ (°C) |
| --- | --- | --- |
| *Il1b* | F: ACACTCCTTAGTCCTCGGCCA | 60 |
|  | R: CCATCAGAGGCAAGGAGGAA |  |
| *Ifng* | F: TGGTGACATGAAAATCCTGCAGAG | 59 |
|  | R: GCTTATGTTGTTGCTGATGGCCTG |  |
| *Lcn2* | F: CAATGTCACCTCCATCCTGGT | 60 |
|  | R: ACTGGTTGTAGTCCGTGGTG |  |
| *Mcj* | F: ACGCCGACATCGACCACACAG | 58 |
|  | R: AATCTTCCTTGCTGTTGCCGTG |  |
| *Myd88* | F: CCGCCTATCGCTGTTCTTGA | 59 |
|  | R: GCCAGGCATCCAACAAACTG |  |
| *Reg3b* | F: TACTGCCTTAGACCGTGCTTTCTG | 60 |
|  | R: GACATAGGGCAACTTCACCTCACA |  |
| *Rpl19* | F: GACCAAGGAAGCACGAAAGC | 60 |
|  | R: CAGGCCGCTATGTACAGACA |  |
| *Timp3* | F: GGCCTCAATTACCGCTACCA | 60 |
|  | R: CTGATAGCCAGGGTACCCAAAA |  |
| *Tlr4* | F: GCAATGTCTCTGGCAGGTGTA | 60 |
|  | R: CAAGGGATAAGAACGCTGAGA |  |
| *Tlr5* | F: ATGGCATGTCAACTTGACTT | 55 |
|  | R: GATCCTAAGATTGGGCAGGT |  |
| *Tlr9* | F: CAGCTAAAGGCCCTGACCAA | 58 |
|  | R: GCGATCCACCGTCTTGAGAA |  |
| *Tnf* | F: AGCCCACGTCGTAGCAAACCAC | 60 |
|  | R: ATCGGCTGGCACCACTAGTTGGT |  |
| *Tnfr1* | F: GCTGTTGCCCCTGGTTATCT | 60 |
|  | R: ATGGAGTAGACTTCGGGCCT |  |
| Conditions | Hold cycle 95°C for 2.30', and then 40x (95°C for 15", 60°C for 1´) | |
| Reagents | PerfeCTa SYBR® Green SuperMix (Quantabio) | |
| Instrument | QuantStudio 6 Flex Real-Time PCR System (Thermo Scientific) | |
| Expression | Normalized to *Rpl19* housekeeping gene using the Pfaffl equation and expressed relative to the mean of a relevant control group | |
